# Predictors of therapeutic response in schizophrenia – preliminary results

**DOI:** 10.1192/j.eurpsy.2023.950

**Published:** 2023-07-19

**Authors:** I. Kamenova, G. Onchev

**Affiliations:** Psychiatry and Medical Psychology, Medical University Sofia, Sofia, Bulgaria

## Abstract

**Introduction:**

Schizophrenia is a heterogenous disease and there is wide variation in the therapeutic response in patients, with some being good responders and others - severely disabled and defined as treatment resistant.

**Objectives:**

To identify specific socio-demographic and clinical characteristics as prognostic factors for therapeutic response in the search of course prognosis and disease outcome.

**Methods:**

The study has naturalistic, non-interventional design and includes one-year prospective follow-up. Schizophrenic patients are being evaluated in three time points – at admission (T1), by discharge (T2), and one year after the hospitalization (T3). Psychopathology is evaluated by the Positive and Negative Syndrome Scale (PANSS), as well as negative symptoms – by the Scale for the Assessment of Negative Symptoms (SANS), aggression by the Modified Overt Aggression Scale (MOAS) and functioning by the GlobalAssessment of Functioning (GAF). Statistical analyses are performed using Descriptive methods, Student’s t-test, Wilcoxon Signed Ranks Test, as well as multiple regression. An ethical approval of the study has been obtained.

**Results:**

The sample consists of 108 patients with mean age of 39 (SD±12.7) and 89.8 % (N=97) of them were prospectively assessed after one year. All symptom dimensions in the 5 -factor model – positive, negative, disorganized, manic, and depressive, measured by PANSS, as well as negative symptoms (objectified by SANS) and aggression (objectified by MOAS) are significantly lower after inpatient treatment. There is an improvement in functioning one year after admission (z=-8.01, p<.001), although both negative symptoms (z=-2,45, p=0.015) and aggressive behavior (z=-4.260, p<.001) are significantly higher one year after discharge. From the multiple regression, at T1, involuntary admission is a significant predictor for higher aggression and lower functioning (p<.001). The duration of hospitalization is longer with lower compliance (p=.022) and the treatment with atypical antipsychotics decreases the hospital stay (p=.021). One year after admission, employment serves as a positive predictive factor as it decreases the psychopathology (p=.001), negative symptoms (p<.001), and improves the functioning (p<.001). Good compliance is a predictor for lower psychopathology (p=.015), less aggression and higher functioning (p<.001).

**Conclusions:**

The inpatient treatment is efficacious in terms of psychopathology, aggression and is linked to better functioning. The naturalistic design shows depletion of the positive effects of treatment in terms of negative symptoms and aggression probably due to incomplete medication compliance, which is a bad prognostic factor for functioning. This implies the need of continuous psychosocial services and better psychoeducation after discharge.

**Disclosure of Interest:**

None Declared

